# PTPN14 aggravates inflammation through promoting proteasomal degradation of SOCS7 in acute liver failure

**DOI:** 10.1038/s41419-020-03014-7

**Published:** 2020-09-25

**Authors:** Beibei Fu, Songna Yin, Xiaoyuan Lin, Lei Shi, Yu Wang, Shanfu Zhang, Qingting Zhao, Zhifeng Li, Yanling Yang, Haibo Wu

**Affiliations:** 1 School of Life Sciences, Chongqing University, 401331 Chongqing, China; 2 Medical School, Yan’an University, 716000 Yan’an, Shaanxi China; 3 Technical Center of Chongqing Customs, 401147 Chongqing, China; 4 Chongqing Center for Disease Control and Prevention, 400042 Chongqing, China

**Keywords:** Acute inflammation, Liver diseases

## Abstract

Acute liver failure (ALF) is a rare but life-threatening systemic disorder. The innate immune regulation has an important role in this process; however, the specific mechanisms are not completely clear. Using the LPS + D-GalN-induced ALF mouse model, we found that the survival rate of PTPN14-deficient mice was higher than that of the control group, while the release of inflammatory factors was significantly lower. We further showed that PTPN14 interacted with SOCS7, and promoted the degradation of SOCS7 through ubiquitination at K11 and K48, thereby reducing the protein level of SOCS7 and weakening the inhibitory effects on inflammatory factors. More importantly, SOCS7 blocked the NF-κB signaling pathway by preventing the activity of the IKK complex, and then reduced the expression of downstream inflammatory factors. In this study, we firstly reported the inhibitory effect of SOCS7 on the NF-κB pathway in the ALF mouse model and elucidated the mechanism of PTPN14–SOCS7–NF-κB axis in the regulation of inflammation. These results provide new insights into the clinical treatment of ALF.

## Introduction

Acute liver failure (ALF) is a rare but life-threatening critical illness, most commonly occurs in patients without preexisting liver disease^1^. Cytokine storm has an important role in the pathogenesis of ALF. The large number of inflammatory cytokines released by the host will attack the liver, leading to severe tissue injury and liver failure^2^. In the face of uncontrolled cytokine storm, steroid treatment is still the main choice of clinical treatment. However, the abuse of steroids produces severe sequelae, greatly reducing patients’ quality of life after recovery^3,4^. Therefore, studying the regulatory mechanisms from a perspective of the innate immune system and identifying key factors that trigger the uncontrolled inflammation, would provide new ideas for the clinical treatment of cytokine storm occurring in ALF.

Protein tyrosine phosphatases (PTPs) are signaling molecules that regulate a variety of cellular processes including cell growth, differentiation, mitotic cycle, and oncogenic transformation^5^. Protein tyrosine phosphatase non-receptor type 14 (PTPN14) in mice consists of 1899 amino acids, including three domains: PTPc-N14, B14, and FERM-C-PTPN14-PTPN21. A wealth of information about the role of PTPN14 in cancer is available^6–8^; however, much less has been reported about its effects on the immune response. A recent study indicated that PTPN14 mediated the dephosphorylation and restoration of vascular endothelial cadherin at adherens junctions in LPS-induced acute lung injury^9^, suggesting that PTPN14 may also have a role in the regulation of inflammation.

Suppressor of cytokine signaling (SOCS) is a family of negative regulatory proteins related to a wide range of cytokines and growth-related factor signals^10^. The SOCS family contains SOCS1-SOCS7 and CIS. They are similar in structure: the middle of the peptide chain contains an SH2 domain and the C terminus has a SOCS box. The SOCS family can bind to receptors on the cell surface. Through cascading reactions of various signal transduction pathways, SOCSs regulate a variety of basic biological processes, such as immune response and individual development^11,12^. As a member of the SOCS family, SOCS7 affects the proliferation of bladder cancer cells by regulating the activity of the JAK-STAT pathway^13^. The deletion of SOCS7 also leads to enhanced insulin action and enlarged pancreatic islets^14^. However, whether or not SOCS7 is involved in the regulation of inflammation has not been elucidated.

In this study, we identified that PTPN14 was pivotal for initiating cytokine storm by using an ALF mouse model. We demonstrated that PTPN14 promoted SOCS7 degradation through ubiquitination at K11 and K48 sites, thereby reducing SOCS7 at the protein level and weakening the inhibitory effects on inflammatory responses. We established a network of PTPN14–SOCS7–NF-κB axis in the regulation of inflammation, which provided a potential drug target for the clinical treatment of ALF.

## Results

### PTPN14-deficient mice show lower inflammation in ALF

**Fig. 1:**
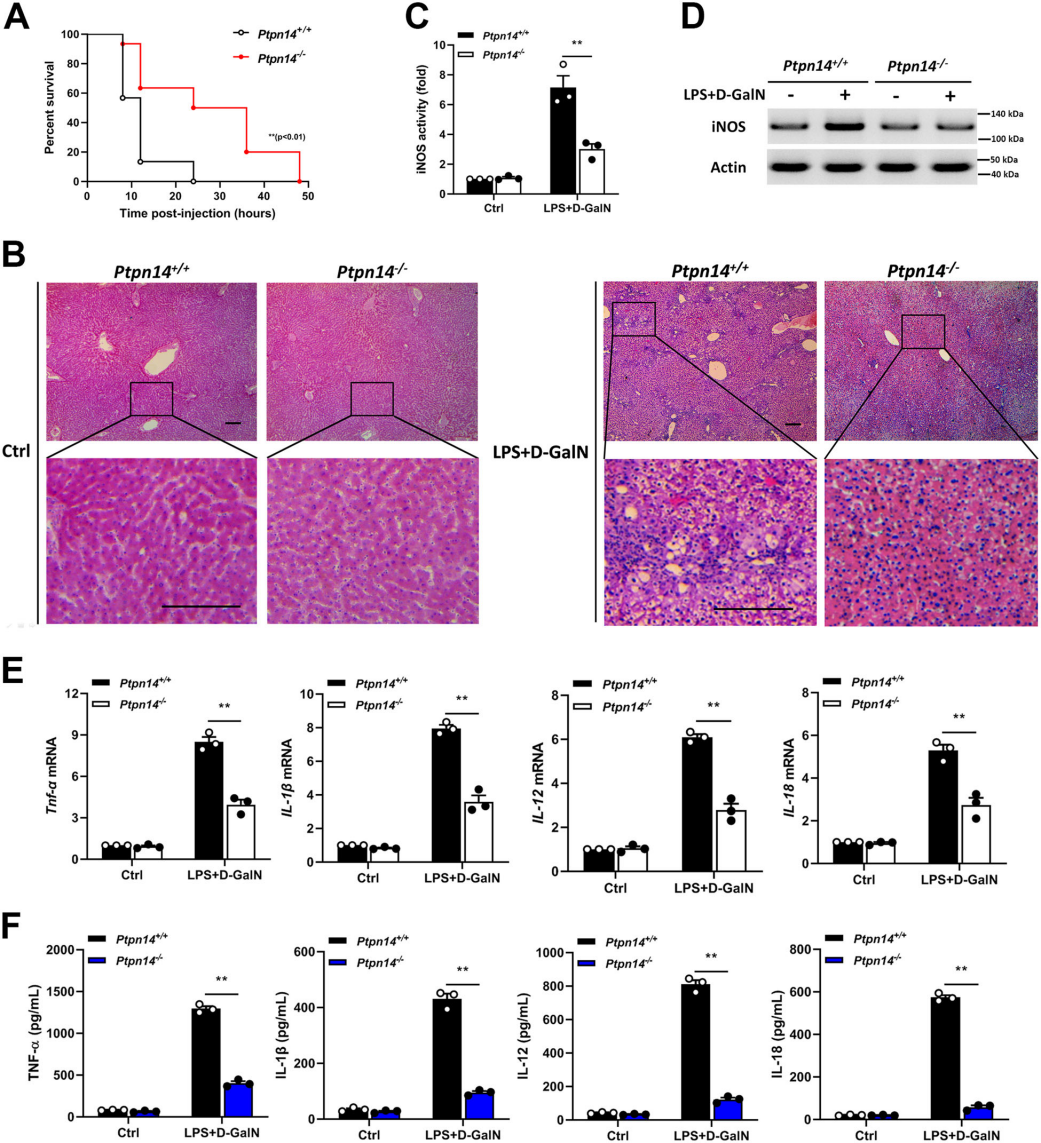
**PTPN14-deficient mice show lower inflammation in acute liver failure.** **a** Wild-type C57BL/6J mice (*n* = 30) and PTPN14-deficient mice (*n* = 30) were injected intraperitoneally with *E. coli* O111: B4 LPS (0.01 mg/kg) and D-GalN (800 mg/kg). Mice were observed for moribundity and lethality within 72 h. **b** At 20 h post-infection, livers were collected for H&E staining to observe the inflammation. Scale bar = 200 μm. **c**, **d** iNOS activity of liver tissue at 20 h post-infection. **e**, **f** qRT-PCR (**e**) and ELISA (**f**) were used to detect the expression levels of TNF-α, IL-1β, IL-12, and IL-18 in the liver. Mouse survival data (**a**) were plotted as Kaplan–Meier curves and compared by log-rank (Mantel–Cox) test. Histopathology (**b**) and blots (**d**) were representatives of three independent experiments. Data shown in **c** and **f** were cumulated from three independent experiments (mean ± s.e.m. of *n* = 3). qRT-PCR data (**e**) were representative of one experiment with at least three independent biological replicates; a single data point represented one technical repeat. Two-tailed Student’s *t*-test was used to compare the means between two groups (**c**–**f**). **p < 0.01.

In order to study the role of PTPN14 in ALF, the wild-type and PTPN14-deficient C57BL/6J mice were stimulated with LPS + D-GalN by intraperitoneal injection to construct an ALF model. The result showed that the survival rate of PTPN14-deficient mice was significantly improved compared to that of the control group (Fig. 1a). At 20 h post-infection, PTPN14-deficient mice showed a slighter liver injury, whereas wild-type mice developed a severe inflammation (Fig. 1b). Through testing the activity of inducible nitric oxide synthase (iNOS), it was found that after LPS + D-GalN stimulation, the activity (Fig. 1c) and protein level (Fig. 1d) of iNOS in PTPN14-deficient mice were much lower than that in wild-type mice (Fig. 1c, d). Then we examined the levels of inflammatory factors in the liver, including TNF-α, IL-1β, IL-12, and IL-18. The results showed that the expression and secretion of inflammatory factors were significantly reduced after PTPN14 knockout (Fig. 1e, f). Taken together, PTPN14-deficient mice showed lower inflammation in ALF, which was beneficial to their survival.

### PTPN14 interacts with SOCS7

**Fig. 2:**
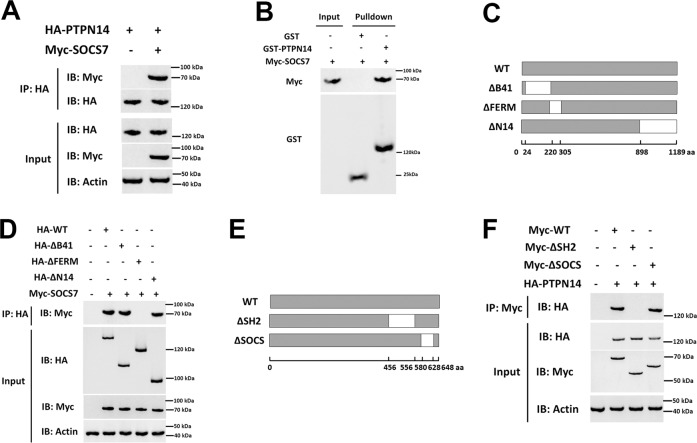
**PTPN14 interacts with SOCS7.** **a** HA-labeled PTPN14 and Myc-labeled SOCS7 were transfected into HEK293T cells, and the interaction between PTPN14 and SOCS7 was detected by Co-IP. **b** The interaction between PTPN14 and SOCS7 was detected by GST pulldown assay in vitro. **c** Schematic diagram of the PTPN14 mutants. **d** The interaction between the PTPN14 mutants and SOCS7 in HEK293T cells was detected using Co-IP. **e** Schematic diagram of the SOCS7 mutants. **f** The interaction between the SOCS7 mutants and PTPN14 in HEK293T cells was detected using Co-IP. All blots were representative of three independent experiments.

To better understand how PTPN14 affects the inflammatory response, we used the yeast two-hybrid to screen the interacting proteins of PTPN14 and found that there was an interaction between SOCS7 and PTPN14. SOCS7 is a member of the SOCS family, which can suppress the expression of inflammatory factors by inhibiting the JAK-STAT pathway^15^. Therefore, we targeted SOCS7 to study the relationship between SOCS7 and PTPN14. The results from coimmunoprecipitation identified the interaction between PTPN14 and SOCS7 (Fig. 2a), and this interaction was confirmed by GST pulldown assay in vitro (Fig. 2b). Next, the mutants of PTPN14 and SOCS7 were constructed and transfected into HEK293T cells. Coimmunoprecipitation was used to study their interacting domains (Fig. 2c, e). The results showed that the FERM domain of PTPN14 and the SH2 domain of SOCS7 were necessary for their interaction (Fig. 2d, f).

### PTPN14 promotes SOCS7 degradation through the ubiquitination at K11 and K48

**Fig. 3:**
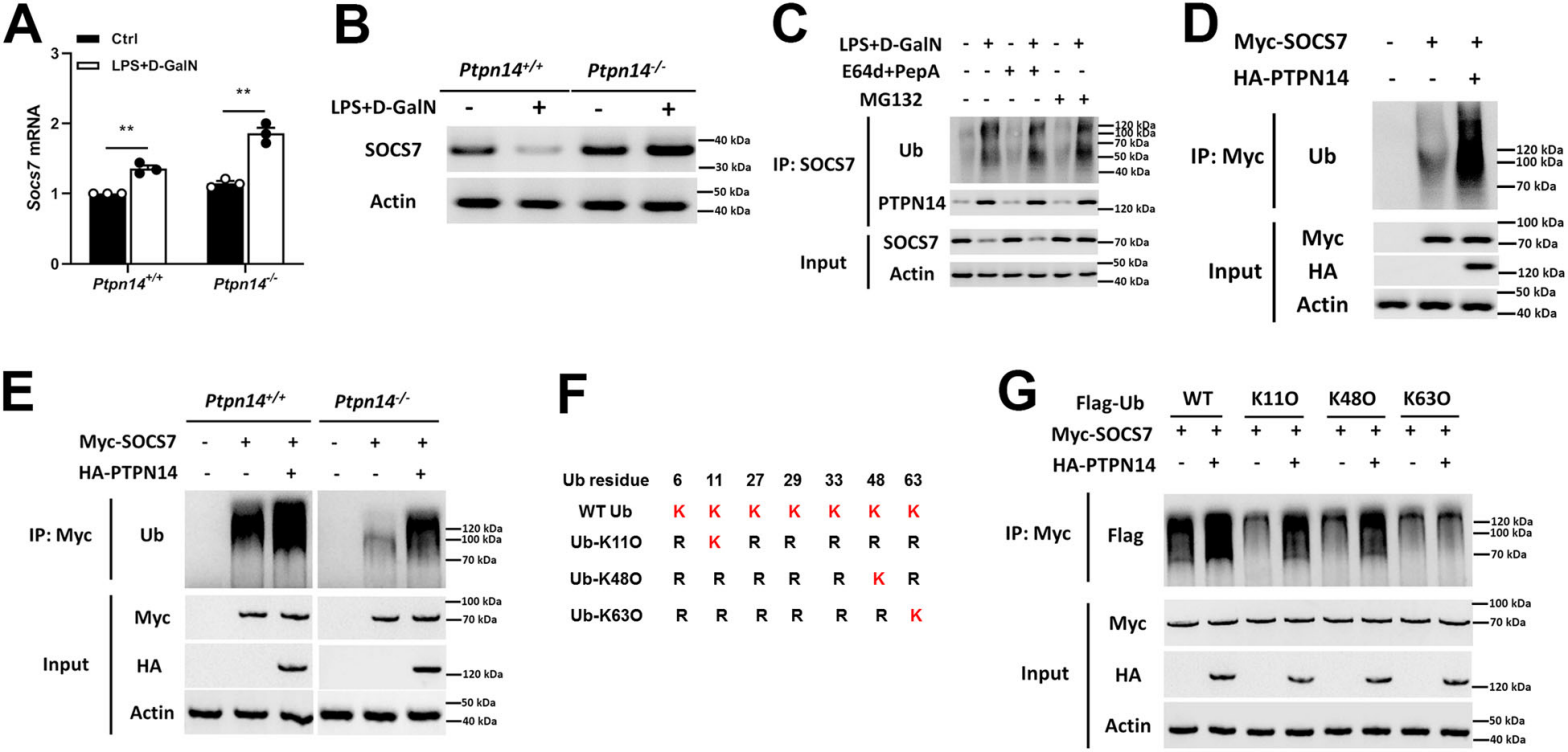
**PTPN14 promotes SOCS7 degradation through ubiquitination at K11 and K48.** **a**, **b** qRT-PCR (**a**) and western blotting (**b**) were used to detect SOCS7 mRNA levels in livers of wild-type and PTPN14-deficient mice stimulated with 0.01 mg/kg LPS and 800 mg/kg D-GalN. **c** Hep G2 cells were treated with E64d+PepA (lysosomal degradation inhibitor) or MG132 (proteasome degradation inhibitor), stimulated with 1 μg/mL LPS and 5 mM D-GalN for 24 h, and then the interaction between SOCS7 and ubiquitin or PTPN14 were detected by Co-IP. **d** HA-labeled PTPN14 and Myc-labeled SOCS7 were transfected into Hep G2 cells. Cells were stimulated with 1 μg/mL LPS and 5 mM D-GalN for 24 h, and then the interaction between SOCS7 and ubiquitin was detected by Co-IP. **e** HA-labeled PTPN14 and Myc-labeled SOCS7 were transfected into wild-type and PTPN14-deficient BMDMs. After stimulation with 1 μg/mL LPS and 5 mM D-GalN for 24 h, the interaction between SOCS7 and ubiquitin was detected by Co-IP. **f** Schematic diagram of the ubiquitin mutants. **g** RAW264.7 cells were transfected with different ubiquitin mutants, HA-labeled PTPN14, and Myc-labeled SOCS7, followed by stimulation with 1 μg/mL LPS and 5 mM D-GalN for 24 h. The interaction between SOCS7 and ubiquitin was detected by Co-IP. In **d**, **e**, and **g**, MG132 was added during cell culture to inhibit the degradation of SOCS7. qRT-PCR data (**a**) were representative of one experiment with at least three independent biological replicates; a single data point represented one technical repeat. Two-tailed Student’s *t*-test was used to compare the means between the two groups. Blots (**b**–**e**, **g**) were representative of three independent experiments. n.s not significant.

On the basis of the interaction between PTPN14 and SOCS7, we further studied the effect of PTPN14 on the expression of SOCS7. The result showed that after LPS + D-GalN stimulation, the mRNA level of *Socs7* increased in both PTPN14-deficient and wild-type mice (Fig. 3a). Interestingly, the changes in SOCS7 protein levels were very different from that in mRNA levels. To be specific, SOCS7 protein was dramatically decreased after LPS + D-GalN treatment in wild-type mice; however, it was increased after LPS + D-GalN treatment in PTPN14-deficient mice (Fig. 3b). These results implied that PTPN14 may be involved in the degradation of SOCS7 protein. Under the circumstance of inhibiting proteasomal degradation by using MG132, we found that PTPN14–SOCS7 interaction promoted the proteasomal degradation of SOCS7 (Fig. 3c). Next, Myc-tagged SOCS7 and HA-tagged PTPN14 were transfected into Hep G2 cells, and coimmunoprecipitation showed that PTPN14 enhanced the ubiquitination of SOCS7 (Fig. 3d). Further, we found that the ubiquitination of SOCS7 was weakened in PTPN14-deficient bone marrow-derived macrophages (BMDMs); however, when PTPN14 was complemented, the ubiquitination level of SOCS7 was recovered (Fig. 3e). Generally, K48-linked chains are a targeting device for protein degradation by the 26S proteasome, whereas K63-linked chains act as molecular scaffolds, bringing together subunits of protein kinase^16,17^. Also, there are studies reported that K11-linked ubiquitin chains act in transcription factor activation^18^. Therefore, we used ubiquitin mutants K11O, K48O, and K63O to study the way of ubiquitin chain linkage of SOCS7 (Fig. 3f). The results showed that PTPN14 promoted the ubiquitination of SOCS7 at K11 and K48, instead of K63 (Fig. 3g). These findings demonstrate that PTPN14 participated in the posttranslational modification of SOCS7, and reduced the content of SOCS7 by promoting ubiquitination.

### SOCS7 blocks the NF-κB pathway by interfering with the assembly of the IKK complex

**Fig. 4:**
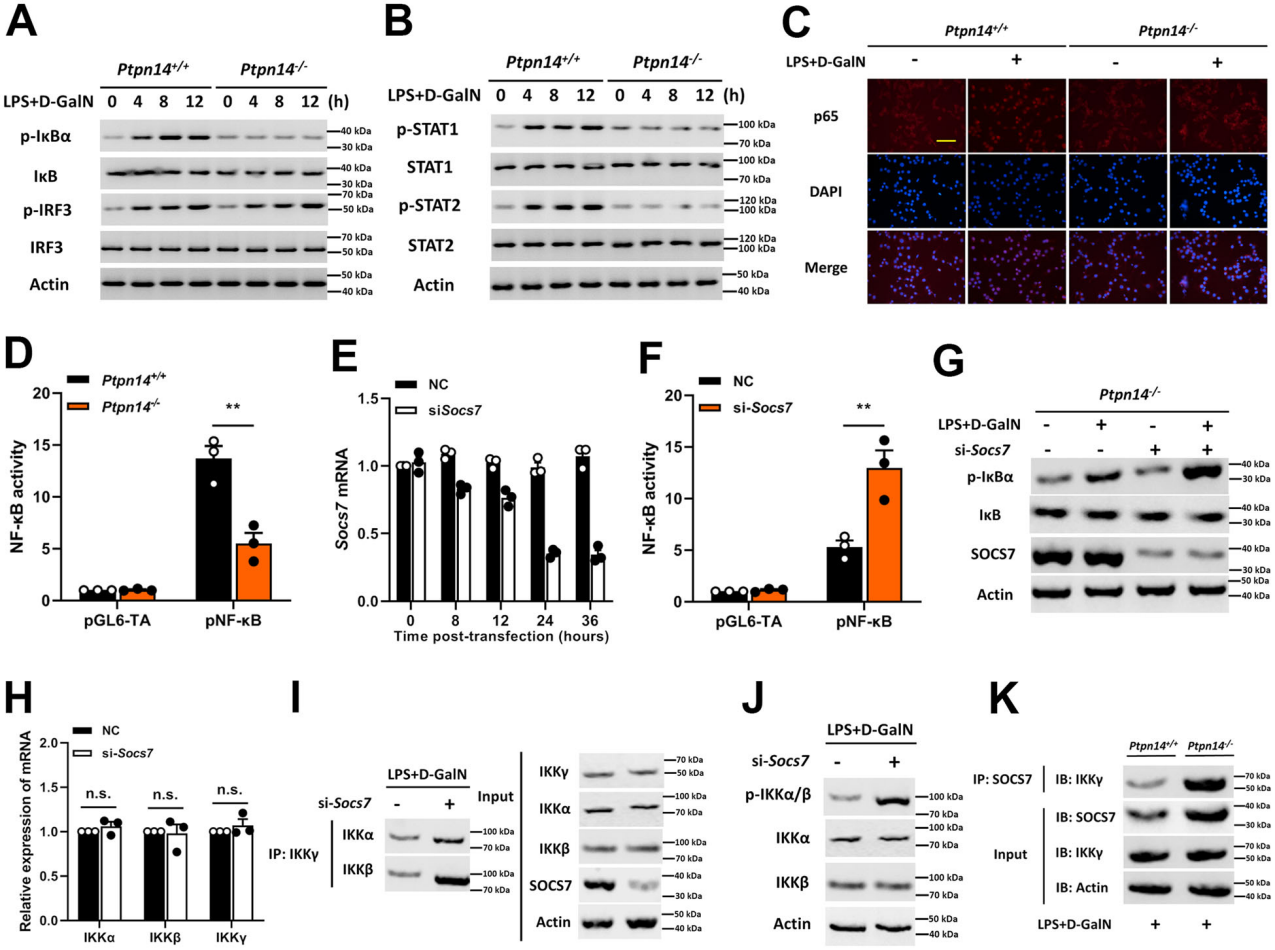
**SOCS7 blocks the NF-κB pathway by interfering with IKK complex assembly.** **a**, **b** Wild-type, and PTPN14-deficient Hep G2 cells were stimulated with 1 μg/mL LPS and 5 mM D-GalN. Cell lysates were collected at time points as indicated and western blotting was used to detect the phosphorylation levels of IκB, IRF-3 (**a**), and STAT1, STAT2 (**b**). **c** Wild-type and PTPN14-deficient Hep G2 cells were stimulated with 1 μg/mL LPS and 5 mM D-GalN for 8 h. The p65 location was identified by immunofluorescence. Scale bar = 50 μm. **d** The activation of the NF-κB pathway in wild-type and PTPN14-deficient BMDMs. **e** The knockdown efficiency of *Socs7* in PTPN14-deficient BMDMs stimulated with 1 μg/mL LPS and 5 mM D-GalN. **f**, **g** PTPN14-deficient BMDMs were transfected with *Socs7* siRNA and were stimulated with 1 μg/mL LPS and 5 mM D-GalN for 24 h. The activation of the NF-κB pathway (**f**) and the phosphorylation level of IκB (**g**) were detected. **h** PTPN14-deficient BMDMs were transfected with *Socs7* siRNA and were stimulated with 1 μg/mL LPS and 5 mM D-GalN for 24 h. The mRNA levels of IKKα, IKKβ, and IKKγ were tested using qRT-PCR. **i**, **j** PTPN14-deficient BMDMs were transfected with *Socs7* siRNA and were stimulated with 1 μg/mL LPS and 5 mM D-GalN for 24 h. The interaction between IKKγ and IKKα or IKKβ (**h**) and the phosphorylation level of IKKα/β (**i**) were detected. **k** Wild-type and PTPN14-deficient BMDMs were stimulated with 1 μg/mL LPS and 5 mM D-GalN for 24 h. The interaction between IKKγ and SOCS7 was detected using Co-IP. Blots (**a**, **b**, **g**, **i**–**k**) and immunofluorescence (**c**) were representatives of three independent experiments. Data shown in **d**, **f** were cumulated from three independent experiments (mean ± s.e.m. of n = 3). qRT-PCR data (**e**, **h**) were representative of one experiment with at least three independent biological replicates; a single data point represented one technical repeat. Two-tailed Student’s *t*-test was used to compare the means between the two groups. **p < 0.01. n.s not significant.

In order to explore the pathways by which PTPN14–SOCS7 complex regulates the expression of inflammatory factors, the phosphorylation levels of IκB, IRF-3, STAT1, and STAT2 were tested in PTPN14-deficient Hep G2 cells following time kinetics. The results showed that after LPS + D-GalN stimulation, the phosphorylation levels of IκB, STAT1, and STAT2 were not significantly increased in PTPN14-deficient hepatocytes (Fig. 4a, b). These indicated that the activation of the NF-κB and JAK-STAT signaling pathways was suppressed by PTPN14 deletion. Since SOCS refers to a family of genes involved in inhibiting the JAK-STAT signaling pathway, the suppression of JAK-STAT signaling can be easily explained^19^. Interestingly, the inhibition of the NF-κB pathway was also observed. In order to further confirm whether PTPN14–SOCS7 complex regulates the NF-κB pathway, we detected the location of p65 in PTPN14-deficient Hep G2 cells after stimulation. The result showed that the deletion of PTPN14 impeded the entry of p65 into the nucleus (Fig. 4c), thus causing the inhibition of the NF-κB pathway (Fig. 4d). Next, *Socs7* siRNA was used to knock down the expression of SOCS7 in PTPN14-deficient cells (Fig. 4e). Compared with untransfected cells, NF-κB activity was restored when *Socs7* was knocked down (Fig. 4f, g). These results showed that PTPN14 promoted the activation of the NF-κB pathway by degrading SOCS7. It was known that the activation of IκB depends on IKK complex^20,21^. We then investigated whether SOCS7 had an effect on the IKK complex activity. The result showed that although SOCS7 had no effect on the expression of IKK components, such as IKKα, IKKβ, and IKKγ (Fig. 4h); the presence of SOCS7 did interfere the interaction between IKKγ and IKKα/β (Fig. 4i), and inhibited the phosphorylation of IKKα/β at the same time (Fig. 4j). Further, the coimmunoprecipitation result showed that SOCS7 had direct interaction with IKKγ (Fig. 4k). Collectively, these data indicated that SOCS7 was abnormally accumulated in PTPN14-deficient cells, and the interaction of SOCS7 and IKKγ prevented the catalytic activity of IKK complex, thus failing to activate IκB and inhibiting the activation of the NF-κB pathway.

### PTPN14–SOCS7 axis regulates the expression of inflammatory factors

**Fig. 5:**
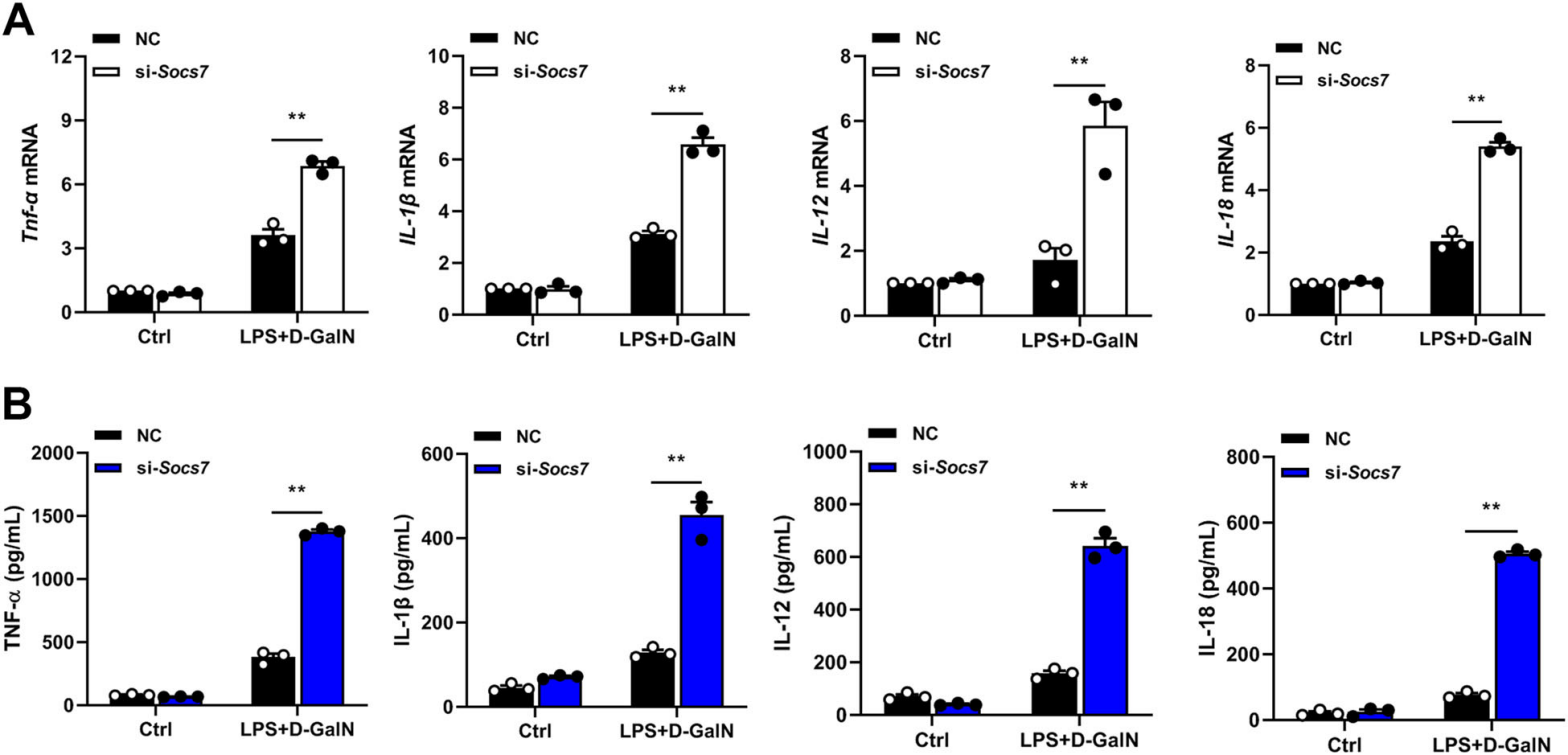
**PTPN14–SOCS7 complex regulates the expression of inflammatory factors.** **a**, **b** PTPN14-deficient BMDMs were transfected with *Socs7* siRNA and were stimulated with 1 μg/mL LPS and 5 mM D-GalN for 24 h. qRT-PCR (**a**) and ELISA (**b**) were used to detect the expression levels of TNF-α, IL-1β, IL-12, and IL-18. qRT-PCR data (**a**) were representative of one experiment with at least three independent biological replicates; a single data point represented one technical repeat. Data shown in **b** were cumulated from three independent experiments (mean ± s.e.m. of *n* = 3). Two-tailed Student’s *t*-test was used to compare the means between the two groups. **p < 0.01.

**Fig. 6:**
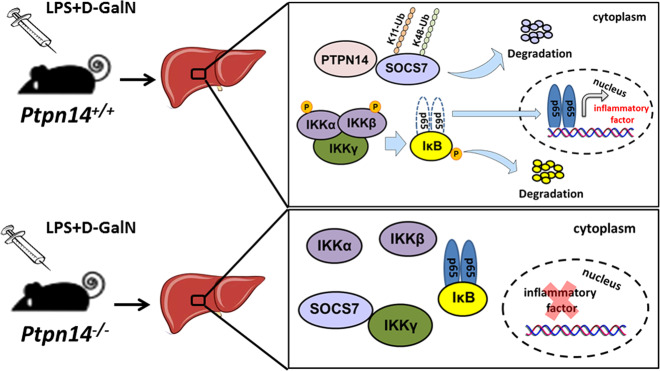
Schematic representation of PTPN14 aggravates inflammation in acute liver failure. In the LPS+D-GalN-induced ALF mouse model, PTPN14 promoted SOCS7 degradation through ubiquitination at K11 and K48. Knockdown of SOCS7 facilitated the assembly of the IKK complex, thereby leading to the activation of NF-κB pathway and the excessive secretion of inflammatory factors.

In order to study the effect of PTPN14–SOCS7 axis on inflammatory cytokines, *Socs7* siRNA was used to knock down the expression of SOCS7 in PTPN14-deficient BMDMs. Inflammatory factors were then detected by real-time quantitative PCR and ELISA. The results showed that the expression levels of inflammatory factors, including TNF-α, IL-1β, IL-12, and IL-18, were restored when SOCS7 was suppressed (Fig. 5a, b). Taken together, PTPN14 negatively regulates SOCS7 (a suppressor of cytokine signaling) by promoting its proteasomal degradation, thus leading to the excessive activation of the NF-κB inflammatory pathway. All these resulted in the formation of a cytokine storm and the severe tissue damage in ALF (Fig. 6).

## Discussion

Cytokine Storm is a phenomenon that produces a large amount of cytokines in a short time and causes severe pathological damage to tissues and organs^22^. The immune damage of cytokine storm is one of the important causes of hepatocyte necrosis in the development of ALF^23^. Cytokine storm in ALF is manifested by the massive secretion of inflammatory factors (such as TNF-α, IL-1β, IL-12, and IL-18), accompanied by necrosis of hepatocytes and infiltration of inflammatory cells in the lobules, and this process is regulated by multiple genes^24^. For example, macrophage inflammatory protein-2 (MIP-2) recruits neutrophils to accumulate in the liver, causing cellular stress, systemic inflammation, and tissue damage and ultimately induces liver failure^25^. Necrotic hepatocytes in ALF release high mobility group box 1 protein (HMGB1) into the extracellular environment, triggering the secretion of pro-inflammatory factors by Kupffer cells. These pro-inflammatory factors further promote the secretion of HMGB1, leading to uncontrolled inflammation^26^. Here we found that a new regulatory gene—PTPN14 was pivotal for initiating the host cytokine storm during ALF. We showed that the deletion of PTPN14 protected SOCS7 from ubiquitin-mediated proteasomal degradation, and accumulation of SOCS7 suppressed NF-κB signaling pathway, thus leading to an inhibition of inflammatory cytokines in ALF mice models. All these data indicated potential targets of PTPN14/SOCS7 for the development of therapeutic approaches for cytokine storm.

Current studies on PTPN14 were mostly focused on cancer. Evidences suggest that in LPS-induced acute lung injury (ALI), phospholipase D2 (PLD2) promotes PTPN14-mediated dephosphorylation of VE-Cadherin and redistribution of VE-cadherin at adherens junctions is essential for the recovery of endothelial barrier function after an edemagenic insult^9^. In this study, the interaction between PTPN14 and SOCS7 was discovered in the liver, and this opened up a pathway to elucidate the immunoregulatory role of PTPN14 in ALF. We found that PTPN14 promoted SOCS7 degradation by ubiquitination through K11 and K48, and regulated the activation of downstream inflammatory pathways. The important role of PTPN14–SOCS7 complex in the regulatory network of inflammation has been established. However, two main questions remain to be addressed: first, are there any posttranslational modifications of SOCS7 catalyzed by PTPN14? As a tyrosine phosphatase, PTPN14 has been shown to form a complex with Yes-associated protein (Yap) and phosphorylate Yap in tumor cells, thereby modulating the Hippo pathway^8^. Existing evidence is insufficient to support that PTPN14 is an E3 ubiquitin ligase, therefore, we hypothesize that PTPN14 may indirectly regulate the ubiquitination degradation process by modifying the phosphorylation of SOCS7; second, how does SOCS7 affect the catalytic activity of IKK complex? In this study, we found that SOCS7 could interact with IKKγ, while the interaction between SOCS7 and IKKα/β could not be observed. We speculate that the presence of SOCS7 will interfere with the assembly of the IKK complex, and IKKα/β complex lacking the IKKγ subunit cannot phosphorylate IκB, resulting in the inhibition of the NF-κB signaling pathway. In addition, we have noticed that the increased phosphorylation of IκBα was not strictly accompanied by the degradation of IκBα, while this did not affect the nuclear entry of p65 and the activation of NF-κB signaling. Therefore, previously undescribed mechanisms of negative feedback regulation in the PTPN14–SOCS7 axis-mediated ALF model may exist, which results in the phenomenon that the total amount of IκBα protein does not decrease with the activation of the NF-κB pathway. However, these hypotheses need to be investigated by further studies.

## Materials and methods

### Ethics statement

This study was carried out in accordance with the Guidelines for the Care and Use of Animals of Chongqing University. All animal experimental procedures were approved by the Animal Ethics Committees of the School of Life Sciences, Chongqing University.

### Mice

6- to 8-week-old C57BL/6J and C57BL/6J-Ptpn14^em1cyagen^ (KOCMP-02378-Ptpn14) mice were purchased from Cyagen Biosciences (Guangzhou, China). All animal study protocols were reviewed and approved by Chongqing University School of Life Sciences review boards for animal studies.

### d-Galactosamine-sensitized LPS challenge

Mice were injected intraperitoneally with *E. coli* O111:B4 LPS (LPS25, Sigma-Aldrich, St. Louis, MO, USA) (0.01 mg/kg) in combination with d-galactosamine (G1639, Sigma-Aldrich) (800 mg/kg)^27^. Mice were observed for moribundity and lethality within 72 h. BMDMs obtained from mice and Hep G2 cells were stimulated with 1 μg/mL LPS and 5 mM D-GalN for 24 h.

### H&E staining

Livers were fixed with 10% buffered formaldehyde for more than 24 h, embedded in paraffin, sectioned, and stained with H&E according to the standard procedure. Photographs were obtained by microscopy (Carl Zeiss, Jena, Germany).

### Cell culture and transfection

HEK293T, Hep G2, and RAW264.7 cells were purchased from American Type Culture Collection (ATCC, CRL-11268, HB-8065, TIB-71). The culture medium was composed of Dulbecco’s Modified Eagle’s Medium (DMEM, Gibco, San Jose, CA, USA) and 10% fetal bovine serum (Gibco). BMDMs were obtained by culturing bone marrow cells as previously described^28^. After 6 days of culture, adherent macrophages were switched into antibiotic-free media and seeded with 10^5^ cells per well. Plasmid DNA and synthetic siRNA were transfected into indicated cells using Lipofectamine 3000 Transfection Reagent (Invitrogen, Life Technologies, CA, USA).

### Plasmids construction

Full-length coding sequences of PTPN14 and SOCS7 (NCBI accession number: NM_008976, NM_138657) were inserted into pCMV-HA, pCMV-Myc, or pGEX-4T-1 vectors. Primers are as follows: HA-PTPN14, F-AACTGTCGACCATGCCTTTCGGC, R-TATAAGCGGCCGCATTGTGTGTAT; Myc-SOCS7, F-AATCGAATTCCGATGCAGGGGG, R-CCCCAGGGCTACAGTCGACTTAA; GST-PTPN14, F-AATCGTCGACCCATGCCTTTCGG, R-TATAAGCGGCCGCATTGTGTGTAT. The WT Ubiquitin and other mutants were purchased from Addgene. The pGL6-TA and pNF-κB used in the measurement of NF-κB activity were purchased from Beyotime (Jiangsu, China).

### Real-time quantitative PCR

Total RNA was isolated using TRIzol reagent (Invitrogen) and the purified RNA was reverse-transcribed using an SYBR PrimeScript RT-PCR Kit (Takara, Otsu, Shiga, Japan) according to the manufacturer’s instructions. The expression of mRNAs was quantified using an SYBR Premix ExTaq II Kit (Takara). Real-time quantitative PCR (qRT-PCR) was performed on an ABI StepOnePlus PCR System (Applied Biosystems, Foster City, CA, USA) and the results were normalized to *β-actin* mRNA levels. Data were analyzed using the 2^−^^ΔΔCt^ method. All primers used for qRT-PCR were purchased from Qiagen (Hilden, Germany).

### Immunoblot and immunoprecipitation

Immunoblot analysis was performed as previously described^29^. Cells were lysed with RIPA buffer (Beyotime). Protein samples were subjected to SDS-PAGE and transferred to PVDF membranes (Millipore, Bedford, MA, USA). Primary antibodies used include 1/1000 anti-Actin (AF5001), 1/1000 anti-GST (AF2299), 1/1000 anti-Flag (AF0036) (Beyotime), 1/1000 anti-HA (SAB2702196), 1/1000 anti-Myc (SAB2702192), 1/500 anti-SOCS7 (SAB2103430), 1/500 anti-IKKγ (SAB3500414) (Sigma-Aldrich), 1/500 anti-iNOS (2982), 1/200 anti-phospho-IκBα (Ser32/36) (9246), 1/500 anti-IκBα (4814), 1/200 anti-phospho-IRF-3 (Ser386) (37829), 1/500 anti-IRF-3 (11904), 1/200 anti-phospho-STAT2 (Tyr690) (4441), 1/500 anti-STAT2 (4594), 1/500 anti-NF-κB p65 (8242), 1/500 anti-IKKα (2682), 1/500 anti-IKKβ (2678), 1/500 anti-phospho-IKKα/β (Ser176/180) (2697) (Cell Signaling Technology, Inc., Danvers, MA, USA), 1/500 anti-Ubiquitin (sc-271289), 1/200 anti-PTPN14 (Pez) (sc-373766) (Santa Cruz Biotechnology, Santa Cruz, CA, USA), 1/500 anti-STAT1 (AHO0832), and 1/200 anti-phospho STAT1 (Tyr701) (44–376G) (Invitrogen). Immunoblots were revealed using the SuperSignal west pico substrate (ThermoFisher Scientific, San Jose, CA, USA). For coimmunoprecipitation (Co-IP), the lysates were incubated with appropriate antibodies and Protein A/G beads (ThermoFisher Scientific) overnight at 4 °C followed by immunoblot analysis.

### Measurement of iNOS activity

The inducible NO synthase (iNOS) activity was detected by the Nitric Oxide Synthase Assay Kit (Beyotime) according to the manufacturer’s instructions. After the d-galactosamine-sensitized LPS challenge, the liver homogenate was treated with 100 μL NOS detection buffer and 100 μL detection reaction solution and then incubated for 30 min at 37 °C. Data were collected with a VICTOR X5 Multilabel Plate Reader (PerkinElmer, Waltham, MA, USA).

### ELISA

Mouse TNF-α, IL-1β, IL-12, and IL-18 ELISA kits were purchased from BD Biosciences (Franklin Lakes, NJ). Cell culture supernatants were assayed according to the manufacturer’s protocols. The concentration of each cytokine was calculated against a standard curve.

### Statistical analysis

All data were from three independent biological experiments with multiple mice and were presented as the mean ± s.e.m. The statistical significance between groups was determined by two-tailed Student’s *t*-test. Mouse survival data were plotted as Kaplan–Meier curves and compared by log-rank (Mantel–Cox) test. A value of *P* < 0.05 was considered significant. GraphPad Prism 8.3 (GraphPad Holdings, San Diego, CA, USA) and SPSS 22.0 (IBM, New York, USA) was used for data analysis.
